# Mapping Breakpoints of Complex Chromosome Rearrangements Involving a Partial Trisomy 15q23.1-q26.2 Revealed by Next Generation Sequencing and Conventional Techniques

**DOI:** 10.1371/journal.pone.0154574

**Published:** 2016-05-24

**Authors:** Qiong Pan, Hao Hu, Liangrong Han, Xin Jing, Hailiang Liu, Chuanchun Yang, Fengting Zhang, Yue Hu, Hongni Yue, Ying Ning

**Affiliations:** 1 Laboratory of Clinical Genetics, Huai’an Maternity and Child Health Care Hospital of Jiangsu Province, Yangzhou University, Huai’an, China; 2 Department of Medical Genetics, Hunan Provincial Maternal and Child Health Hospital, Changsha, China; 3 Department of Pediatrics, Huai’an Maternity and Child Health Care Hospital of Jiangsu Province, Huai’an, China; 4 CapitalBio Genomics Co., Ltd, Dongguan, China; 5 BGI-Shenzhen, Shenzhen, China; University of Bonn, Institute of Experimental Hematology and Transfusion Medicine, GERMANY

## Abstract

Complex chromosome rearrangements (CCRs), which are rather rare in the whole population, may be associated with aberrant phenotypes. Next-generation sequencing (NGS) and conventional techniques, could be used to reveal specific CCRs for better genetic counseling. We report the CCRs of a girl and her mother, which were identified using a combination of NGS and conventional techniques including G-banding, fluorescence in situ hybridization (FISH) and PCR. The girl demonstrated CCRs involving chromosomes 3 and 8, while the CCRs of her mother involved chromosomes 3, 5, 8, 11 and 15. HumanCytoSNP-12 Chip analysis identified a 35.4 Mb duplication on chromosome 15q21.3-q26.2 in the proband and a 1.6 Mb microdeletion at chromosome 15q21.3 in her mother. The proband inherited the rearranged chromosomes 3 and 8 from her mother, and the duplicated region on chromosome 15 of the proband was inherited from the mother. Approximately one hundred genes were identified in the 15q21.3-q26.2 duplicated region of the proband. In particular, *TPM1*, *SMAD6*, *SMAD3*, and *HCN4* may be associated with her heart defects, and *HEXA*, *KIF7*, and *IDH2* are responsible for her developmental and mental retardation. In addition, we suggest that a microdeletion on the 15q21.3 region of the mother, which involved *TCF2*, *TCF12*, *ADMA10* and *AQP9*, might be associated with mental retardation. We delineate the precise structures of the derivative chromosomes, chromosome duplication origin and possible molecular mechanisms for aberrant phenotypes by combining NGS data with conventional techniques.

## Introduction

Complex chromosomal rearrangements (CCR) are rare, structural chromosomal abnormalities involving three or more breakpoints located on two or more chromosomes [1]. Individuals with CCRs can be phenotypically normal or display a clinical abnormality. These abnormalities result from microdeletions or microduplications near the translocation breakpoints, disruption of the genes located in the breakpoints or elsewhere in the genome, or position effect [2].

Conventional cytogenetic analysis is limited in determining whether a CCR is balanced or unbalanced. Fluorescence in situ hybridization (FISH) techniques can dissect complex chromosomal aberrations and reveal heterogeneity while also providing a general view of the whole karyotype [3]. The recent application of genome-wide microarrays, comparative genomic hybridization arrays (CGH-arrays) and single-nucleotide polymorphism arrays (SNP arrays) in patients with an abnormal phenotype has detected submicroscopic imbalances that could be associated with a particular disease [4,5]. However, because of the relatively low resolution of these traditional techniques, they can, at most, detect microdeletions or microduplications at approximately 10 Kb and cannot characterize the specific breakpoints on the genome, although this limitation may be offset by next-generation sequencing (NGS) [6]. However, the coverage rate of several points of the genome may lead to the ignoring of some rearrangements. Thus, the combination of several molecular cytogenetic techniques with NGS contributes to the identification of more chromosomal breakpoints or genomic imbalances, thereby providing greater insight into the complexity of the CCRs. It has been reported that NGS was applied in the study of balanced chromosome rearrangements [7,8], and recently, an integration of traditional cytogenetic techniques with NGS was used to delineate structural chromosome rearrangements at the nucleotide level [9].

Distal trisomy 15q is an extremely rare chromosomal disorder involving duplication of the distal portion of the long arm on chromosome 15, which was first reported in 1974 by Fujimoto et al [10]. In most cases, distal trisomy 15q is due to a chromosomal unbalanced translocation in one of the parents [11], and de novo trisomy cases are rarely reported [12,13]. This disorder is characterized by growth delays before and/or after birth (prenatal and/or postnatal growth retardation), mental retardation, and/or distinctive malformations of the craniofacial area, among others [12]. In addition, duplication of 15q11.2-q13 has been associated with autism [14]. The range and severity of symptoms and physical findings vary from case to case, depending upon the length and location of the duplicated portion of chromosome 15q.

15q21 microdeletion syndrome is considered a well-defined and clinically recognizable phenotype [15]. The common clinical phenotypes include mental retardation, growth retardation, a beak-like nose with hypoplastic alae nasi and a thin upper lip. The common deletions of chromosome 15 have been previously reported to involve the interstitial region encompassing bands 15q21-q22 [16].

In the present study, we report a 35.4 Mb 15q21.3-q26.2 duplication in the proband and a 1.6 Mb 15q21.3 microdeletion in her mother. CCRs in the proband and her mother were revealed by the combination of G-banding, SNP array, FISH, NGS and PCR.

## Materials and Methods

### Subject Recruitment

The proband, a female neonate of Han people from Jiangsu in China, was born by uterine-incision delivery after pregnancy (G1P1) at 38^+2^ weeks of gestation because of oligohydramnios, intra-uterine asphyxia, and foetal growth restriction. Her mother was 31 years old with intellectual disability and her father was healthy in 44 years old. The birth parameters included the following: birth weight 1,730 g, birth length 44 cm, head circumference 28 cm, and Apgar score 7-8-10 at 1, 3, and 5 minutes, respectively. Physical examination after birth showed left high-parietal bone, patent anterior fontanelles, sagittalis suture separate 3 cm, low flat nose, high arched palate, palpebral fissures slightly oriented downwards, long prominent philtrum, small jaw (micrognathia) and tapering fingers (S1 Fig). She was diagnosed with atrial septal defect (ASD), patent ductus arteriosus (PDA) and pulmonary hypertension by two-dimensional color Doppler echocardiography. She was observed to have developmental delays by her father at nearly six month after her birth. At 6 months, her weight was 3850 g, length 56 cm, head circumference was 36.7 cm, and anterior fontanels measured 4.5×4.5 cm. She was diagnosed with hydrocephalus by cerebral computed tomography (CT) brain scan at 6 months. At 10 months, She could not sit, climb or turn over, and she had no teeth.

The proband’s mother was born from healthy non-consanguineous parents. She was the oldest daughter and had a healthy younger brother who died of drowning. The karyotypes of her parents were not available. She was a girl with intellectual disability with speech difficulties and was unable to communicate; she had a dull expression and an old face, but no history of seizure (S1 Fig). Her general health was normal.

This study was approved by the Medicine Ethics Committee of Huai’An Maternity and Child Health Care Hospital. A written informed consent was obtained from the father of the proband for further analysis, publication of photographs and clinical information.

### Cytogenetic analysis

Chromosome analysis was performed on peripheral blood of the proband and her parents using a conventional G-Banded technique with a resolution of approximately 400 bands. A total of 5 mL of peripheral blood was collected from each member of the family. All samples were subjected to a lymphocyte culture according to a standard cytogenetic protocol. Twenty metaphase cells each were analyzed for the proband and her parents.

### SNP array analysis

The screening of genomic rearrangements and mapping of the breakpoints were performed with the HumanCytoSNP-12 BeadChip platform (Illumina), comprising 301,232 SNPs. Genomic DNA was prepared from peripheral blood and then extracted using a DNeasy Blood & Tissue Kit (Qiagen) according to manufacturer’s protocol. Genomic DNA samples were adjusted to a final concentration 50 ng/μL. DNA amplification, tagging and hybridization were performed according to the manufacturer's protocol. Array slides were scanned on the iScan Reader (Illumina). The GenomeStudio V2011 software (Illumina) was used to analyse the genotypes (human genome build 37/Hg19 for analysis) and evaluate the experimental quality. The call rates of the samples were greater than 99.5%.

### FISH analysis

In order to determine the positions of the chromosome breakpoints involved in the rearrangements on the derivative chromosomes, FISH analysis was performed for the mother using specific probes (Vysis) according to the manufacturer's instructions. The following DNA probes were selected: telomeric probes specific for the chromosomes 3, 5, 8 pter (green signal) and chromosomes 3, 5, 8, 15 qter (red signals) regions (Vysis); D5S23 on 5p15 (green); EGR1 on 5q31(red); ETO on 8q22 (red); MLL (SCN4B/TREH) on 11q23; D15Z1 on 15p11.2 (aqua); SNRPN on 15q12 (red), PML on 15q24.1 (green); AML1 on 21q22 (green).

### Sequencing and bioinformatics analysis

The procedure for sample preparation and sequencing, alignment and filtering, and detection of translocations was performed as previously described by Dong et al [17]. The detailed methods are provided in the S1 File.

### Verification of chromosomes rearrangements by PCR and Sanger sequencing

Five primers were designed to verify NGS results. The primer sequences are shown in S1 Table. The junction fragments were amplified by Platinum^®^ PCR SuperMix High Fidelity regent (Invitrogen) according to the following protocol. First, 27 μL Platinum^®^ PCR SuperMix High Fidelity regent, 10 ng DNA, 10 pM forward primer and 10 pM reverse primer were combined in a volume of 30 μL. For primers Chr11-5r, Chr15-15, Chr5-5r, Chr5r-5, the reaction mixture was incubated for 5 min at 95°C followed by 35 cycles of denaturation for 30 s at 95°C, hybridization for 30 s at 60°C, and elongation for 1.5 min at 72°C, with a final elongation phase for 10 min at 72°C. For primer Chr3-15r, the reaction mix was incubated for 5min at 95°C followed 35 cycles of denaturation for 15 s at 95°C, hybridization for 30 s at 55°C, and elongation for 1.5 min at 72°C, with a final elongation phase for 10 min at 72°C. Then PCR products were sequenced by Sanger method (Invitrogen).

## Results

### The G-banding karyotype of the whole family of the proband

**Fig 1:**
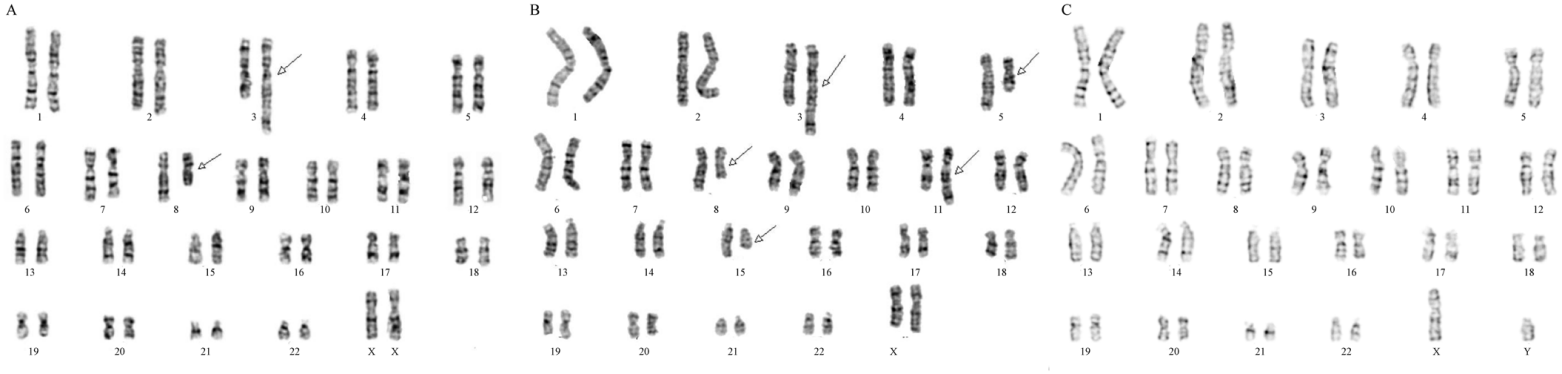
Chromosome G-banding karyotype showing a complex chromosome rearrangement of the proband and her mother. (A) Aberrant chromosomes 3 and 5 of the proband. (B) CCRs between chromosomes 3, 5, 8, 11 and 15 of the mother. (C). Normal karyotype of the father. Arrows point to derivative chromosomes.

G-banding analysis of the karyotype is widely used to detect chromosome structure variants [18,19]. Karyotype of the proband and her parents were analyzed by G-banding at the 400-band level, showing a complex chromosomal rearrangement (CCRs) involving chromosomes 3 and 8 in the proband (Fig 1A) and CCRs involving 5 chromosomes (3, 5, 8, 11 and 15) in the mother (Fig 1B), including two types of CCRs [20]: an exceptional CCR containing multiple breakpoints with insertions among chromosome 3, 8 and 15; a CCR of reciprocal translocation between chromosome 5 and 11 with inversion of segments in derivative 5. The proband acquired derivative chromosomes 3 and 8 from her mother and carried a duplication in chromosome 15. The karyotype in the father is normal (Fig 1C).

### SNP array analysis of the microdeletions and microduplications of the proband and her mother

**Fig 2:**
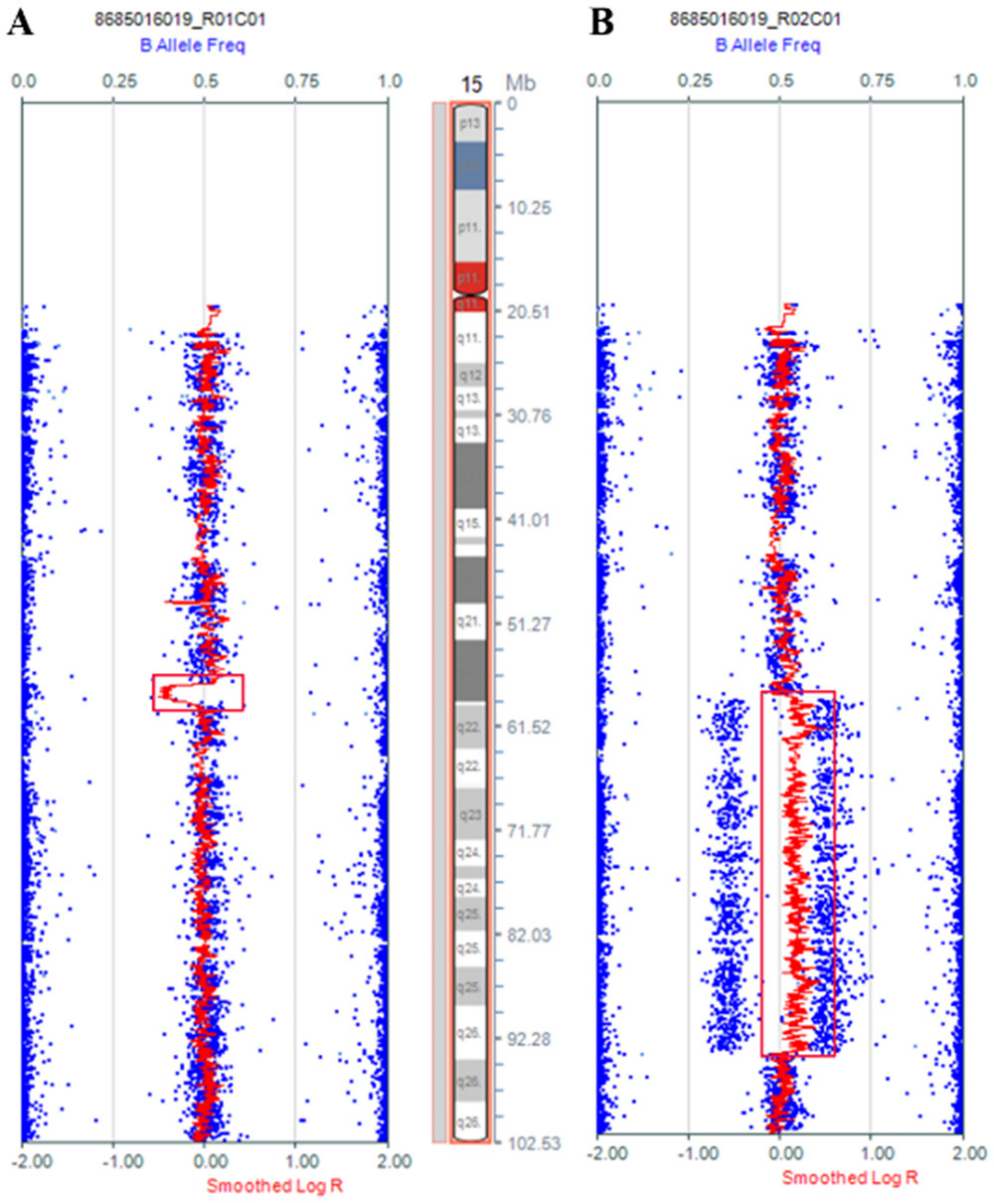
Illumina SNP array results for the distal 15q region. (A) Proband's mother had a 1.6 Mb microdeletion at the 15q21.3 region (chr15: 57413776–59084268) (Human GRCh37/hg19 Assembly). (B) The Proband had a 35.4 Mb microduplication at the 15q21.3-q26.2 region (chr15: 58914326–94371025) (Human GRCh37/hg19 Assembly).

Copy number variations (CNVs) are usually analyzed by SNP array with several software packages [21]. Due to the resolution limitation of G-banding analysis, more CNVs were detected by SNP array using KaryoStudio Software. For the proband and her mother had abnormal phenotypes and a complex translocation involving several breakpoints, the gain or loss of chromosomal materials may be missed by conventional cytogenetic analyses. All CNVs and genes involved chromosome rearrangements were listed in Table 1. A 35.4 Mb duplication on 15q21.3-q26.2 (chr15: 58,914,326–94,371,025, Hg19, NCBI build 37) of the proband was found (Fig 2). Additionally, analysis of her mother showed a 1.6 Mb microdeletion in the 15q21.3 region from chr15: 57,413,776 to chr15: 59,084,268 (Fig 2). In addition, chromosome 5 was broken at 5q21.1, with a 0.5 Mb microdeletion on the 5q21.1 region from chr5: 101,662,158–102,172,751, containing only the RefSeq gene SLCO6A1 (S2 Fig). Based on the array data of the mother, the break regions of the chromosome were at 11p11 and 11q11 (S2 Fig). However, no significant CNVs were seen on chromosomes 3 or 8 (S2 Fig).

**Table 1:** Analysis of genes involved in the variation region on different chromosome.

Case	CNV’s coordinates	Size(Kb)	Gain/Loss	Band	Gene
**Patient**	Chr15: 58914326–94371025	35456	3 copies	q21.3-q26.2	*TPM1;SMAD6*;*SMAD3*;*HCN4*;*MEK1*;*HEXA*;*HEXB*;*KIF7*;*ADAM10*;*HSP90AB4P*;*FAM63B*;*SLTM*;*CCNB2*
	Chr19: 20619061–20722228	103	0 copy	p12	*ZNF737*
	Chr1: 148889571–149728859	839	3 copies	q21.2	*FCGR1C*;*HIST2H2BF*;*PPIAL4B*
**Mother**	Chr15: 57413776–59084268	1670	1 copy	q21.3	*TCF12*;*CCNL1*;*GCOM1*;*MYZAP*;*ALDHTA2*;*AQP9*;*ADAM10*;*FAM63B*;*LOC283663*;*CGNL1*;*MYZAP*;*POLR2M*;*LIPC*;*HSP90AB4P*; *POLR2M*
	Chr19: 20619061–20722228	103	0 copy	p12	*ZNF737*
	Chr22: 39365776–39392296	26	0 copy	q13.1	*APOBEC3B*
	Chr5: 101662158–102172751	510	1 copy	q21.1	*SLCO6A1*
	Chr11: 54835623–56269410	1433	3 copies	q11 q12.1	*TRIM48*
	Chr11: 48331739–50268799	1937	3 copies	p11.2 p11.12	*FOLH1*

### FISH analysis

**Fig 3:**
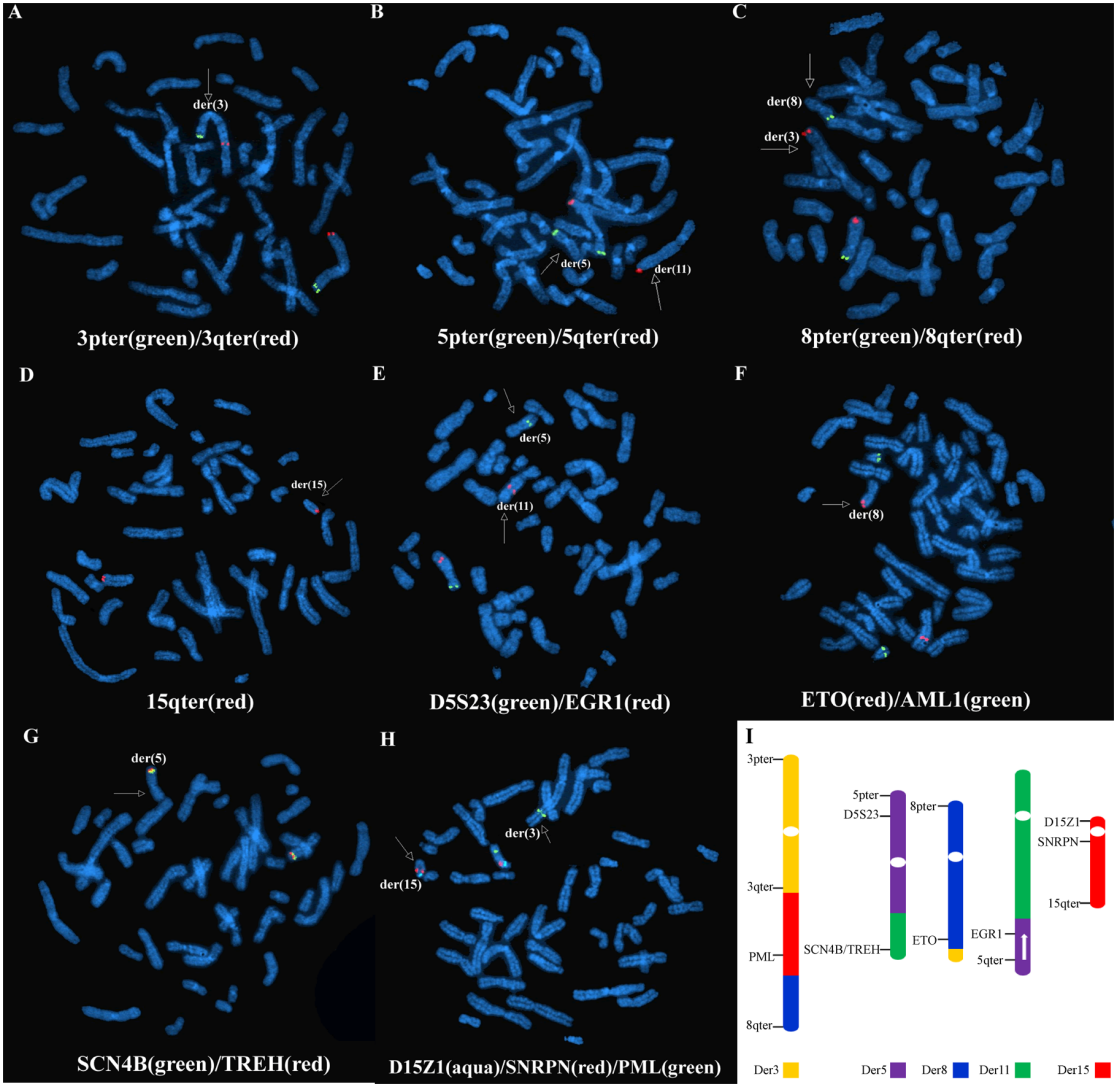
Representative images from FISH analysis of the proband’s mother. (A) FISH using the Telomere 3p probe (spectrum green) and the Telomere 3q probe (spectrum red) revealed a green and red signal on der(3), indicating that the bottom of der(3) was the region of the translocation chromosome. (B) FISH using the Telomere 5p probe (spectrum green) and the Telomere 5q probe (spectrum red) revealed the absence of the red signal and presence of the green signal on der(5), as well as the presence of the red signal on der(11), indicating that telomere 5q was translocated to chromosome 11. (C) FISH using the Telomere 8p probe (spectrum green) and the Telomere 8q probe (spectrum red) revealed the absence of the red signal and presence of the green signal on der(8), as well as the presence of the red signal on der(3), indicating a translocation of 8q to chromosome 3. (D) FISH using the Telomere 15q probe (spectrum red) showed the presence of the red signal on der(15), indicating subtelomeric 15q sequences were retained on der(15) with a subterminal deletion on der(15). (E) FISH using probe D5S23 (spectrum green) at 5p15 and the probe EGR1 (spectrum red) at 5q31 revealed the absence of the red signal and presence of the green signal on distal der(5)’ short arm, as well as the presence of the red signal on der(11), indicating an inversion on the distal short arm of der(5)’. (F) FISH using the probe ETO (spectrum green) at 8q22 and the probe AML1 (spectrum red) at 21q22 showed the presence of the red signal on der(8) and the presence of the green signal on 21, indicating a breakpoint on chromosomes 8q22; the 22q regions of 21 were normal.(G)The MLL probe SCN4B (spectrum green) and TREH (spectrum red) at 11q23 showed the presence of the green and red signal on der(5), indicating a reciprocal translocation between chromosomes 11q and 5q. (H) FISH using probe D15Z1 (spectrum aqua) at 15p11.2, SNRPN (spectrum red) at 15q12 and PML (spectrum green) at 15q24.1 showed the presence of the aqua and red signal on der(15), as well as the presence of the green signal on der(3), indicating a reciprocal translocation between chromosomes 15q and 3q. (I) Probes used with FISH for the detection of chromosome breakpoints as well as CCRs. Reversed arrow demonstrates an inversion region.

**Fig 4:**
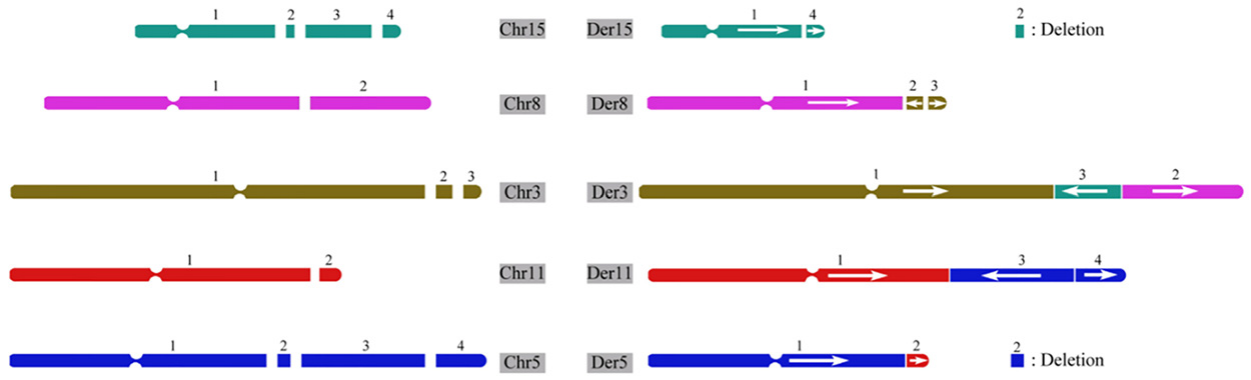
Sequencing results of the CCRs of the mother. Partial karyotype and ideogram of the proband’s mother showed the exceptional CCRs between chromosomes 3, 5, 8, 11 and 15. Reversed arrow indicates chromosome inversion.

To further analyze the complex chromosome structure variations, FISH data from the mother was performed using several probes. On the der(3), telomere analysis for chromosome 3 was normal, but the 8q telomere was located on the distal long arm of the der(3) (Fig 3A and 3C), and probe ETO (at 8q22) was located on the distal long arm of the der(8) due to the t(3;8) (q29;q21) (Fig 3F). Probes for the chromosome 15 including probe PML (at15q24.1), probe D15Z1 (15p11.2) and probe SNRPN (15q12) hybridized to the der(3), demonstrating an insertion of 15q21.3-q26.2 into the long arm of the der(3) (Fig 3H). On the der(5), telomere analysis showed that the 5p probe hybridized normally, but the 5q telomere was located on the distal long arm of the der(11) (Fig 3B), and the SCN4B probe (at 11q23)/TREH (at 11q23) demonstrated that the 11q telomere was located on the distal long arm of the der(5) (Fig 3G). The karyotype and probe demonstrated a t(5;11)(q21;q23). The probe D5S23 (at 5p15) indicated an inversion on the der(5) (Fig 4E). Together, the characterized chromosome rearrangements are demonstrated in Fig 3I.

### Designated karyotypes of the proband and her mother

Thus, on the basis of conventional and molecular cytogenetic analysis, the mother's karyotype was designated as follows: 46, XX, ish der(3)t(3;8)(q29;q21)ins(8;15)(q22;q21.3q26.2)(3pter+,3qter+,SNRPN-, PML+,8qter+), ish der(5) inv(5)(p15.3q14.3)t(5;11)(q21;q23)(5pter+,D5S23+,MLL+), ish der(8) t(3;8)(q29;q21)(8pter+,ETO+,8qter-), ish der(11)t(5;11)(q21;q23)(5qter+), der(15)del(15)(q21.3 q26.2)(15qter+,D15Z1+,SNRPN+). arr5q21.1(101662158–102172751)x1, 15q21.3(57413776–59084268)x1. In addition, the proband's karyotype was designated as 46,XX,der(3)t(3;8)(q29;q21)ins(8;15)(q22;q21.3q26.2)mat,der(15)dup(15)(q21.3q26.2),arr15q21.3q26.2(58914326–94371025)x3.

### Sequencing results of the whole family

Karyotyping, array-CGH (aCGH) and SNP arrays are useful tools to detect chromosome abnormalities. However, balanced translocation and inversion can only be detected through karyotype observation at a low resolution of approximately 5 Mb, whereas these cannot be identified by aCGH and SNP arrays because of the loss of CNVs. Next, we explored the genotype-phenotype relationship using NGS, which has successfully characterized chromosome variations, such as micro-insertions and inversions, on the nucleotide level [22]. In this family’s case, we employed our in-house approach for each subject independently [17]. According to the NGS results, no breakpoint was noticed in the proband’s father (data not shown), whereas twelve breakpoints were involved in the CCRs of the proband’s mother (S3 Fig). The complex rearrangement linkage paragraph is shown in S3 Fig. A 1.68 Mb and a 530 Kb deletion were identified on chromosomes 15 and 5, respectively. Also, 4 inversions on the derivative chromosomes 3, 5, 8 and 11 were revealed by NGS (Fig 4). The proband’s abnormal der(8) and der(3) were inherited from her mother. Genes located at the breakpoints are also marked in S3 Fig.

**Fig 5:**
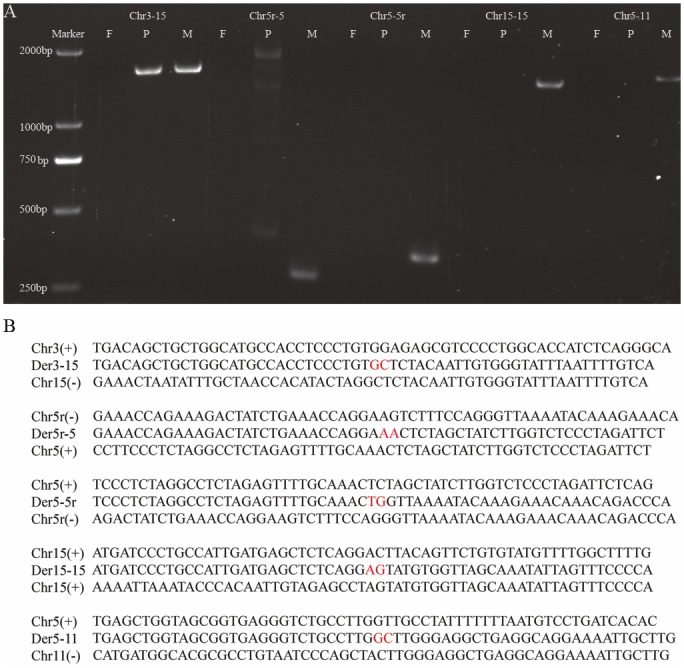
PCR confirmation of chromosome arrangements of the proband and her parents. (A) PCR results of chromosome rearrangements of Chr3-15, Chr5r-5, Chr5-5r, Chr15-15 and Chr5-11 of the proband and her mother. The normal father was regarded as a control. (B) The sequence results of the rearrangement chromosomes using the Sanger method. Each three lines represent one chromosome; the first line and the third line show the normal chromosome sequence; and the middle line demonstrates the rearrangement chromosome sequence, according to the specific regions of chromosome rearrangements shown in Fig 4. Chr3-15: combination of region 1 of chromosome 3 and region 3 of chromosome 15; Chr5r-5: combination of region 2 and region 3 of chromosome 5; Chr5-5r: combination of region 1 and region 2 of chromosome 5; Chr15-15: combination of region 1 and region 4 of chromosome 15; Chr5-11: combination of region 5 of chromosome 11 and region 5 of chromosome 5. F: father, P: proband, M: mother. 5r: reversed region 2 of chromosome 5. Der: derivative sequence of rearrangement chromosome. The red nucleotides represent the specific sites of translocation breakpoints.

The specific regions are shown in Fig 5, including Chr11-5r for region 5 of chromosome 11 and region 5 of chromosome 5; Chr15-15 for region 1 and region 4 of chromosome 15; Chr5r-5 for region 1 and region 2 of chromosome 5; Chr5r-5 for region 2 and region 3 of chromosome 5; Chr3-15r for region 1 of chromosome 3 and region 3 of chromosome 15.

### PCR analysis and Sanger sequencing of breakpoints and chromosome rearrangements

In addition, to confirm the results of the bioinformatics analysis of the chromosome arrangements of the proband and her mother, we chose five breakpoints and designed five primers. Specifically, according to Fig 5, a combination of region 5 of chromosome 11 and region 5 of chromosome 5, region 1 and region 4 of chromosome 15, region 1 and region 2 of chromosome 5, as well as region 2 and region 3 of chromosome 5 was examined by PCR (Fig 5A). These rearrangements were only observed in the mother and were not found in the proband or her father. As G-banding analysis showed that the proband inherited chromosome 3 from her mother, we designed a primer for verifying the combination between region 1 of chromosome 3 and region 3 of chromosome 15. We detected specific rearrangement of these two regions in the proband and her mother, but not in the father (Fig 5A). The PCR products were purified and sequenced with a 3730 DNA analyzer, and the sequence information of each rearrangement is shown in Fig 5B and three of five showed have 1–3 nucleotide microhomologies.

## Discussion

CCRs are usually identified with traditional cytogenetic G-banding analysis. However, the low resolution of this approach prevents the precise identification of breakpoints in the genome. Thus, array-based techniques and FISH are applied for detailed characterization of CCRs [23,24]. Furthermore, NGS is capable of refining the traditional methods on the nucleotide level. In this study, G-banding analysis, SNP array and FISH, combined with NGS, were used to explicitly illustrate the CCRs of a proband and her mother. PCR was used to confirm the results of the rearrangement analysis. Although G-banding, SNP array and FISH identified some balanced rearrangements, in combination with NGS, additional inversions and microdeletions were found. Loss of genetic material leads to complex imbalanced rearrangements; as a result, there is a higher probability of abortion, infant death, and abnormalities in the case of imbalanced gametes conjugating with normal gametes. Thus, strict alignment was applied to acquire accurate results. In fact, to some extent, loosening the alignment may identify more breakpoints [6], which should be tested in subsequent analysis.

In this study, the G-banding karyotype revealed CCRs of the proband and her mother, and the proband inherited derivative chromosomes 3 and 8 from her mother (S1 Fig). A 35.4 Mb duplication on 15q21.3-q26.2 of chromosome 15 of the proband and a 1.6 Mb microdeletion on the 15q21.3 region from chromosome 15 of the mother were characterized using HumanCytoSNP-12 Chip analysis (Fig 3). FISH and PCR were applied to determine the positions of the chromosome breakpoints and chromosome rearrangements (Fig 4). Our NGS data explicitly characterized the specific breakpoints and linkage patterns at the nucleotide level (Fig 5 and S3 Fig).

The mother, with severe mental retardation, had two types of complex chromosome structural rearrangements involving an exception CCR with insertions among chromosomes 3, 8, and 15, and a CCR with reciprocal translocation between chromosomes 5 and 11 with inversion of segments in derivative 5. Since the CCRs involved several chromosomes, it may result from chromothripsis during the pachytene stage of meiosis I [25]. Rearrangements of great complexity will lead to great potential of abnormal gametes and increase the proportions of complicated karyotypes in offspring [26].

With the exception of distal inverted duplication of 15q (q21.3-q26.3) as described in Italian patients [27], and to the best of our knowledge, 15q21.3-q26.2 duplication has not been reported in the cytogenetics database, Online Mendelian Inheritance in Man database, Chromosomal Variations in Man database, Decipher database or the database of Chinese human chromosomal abnormal karyotypes. Considering that the mother had a normal and partially deleted chromosome 15 and the father had a normal chromosome 15, the abnormal trisomy of chromosome 15 of the proband resulted from de novo duplication. According to the SNP array data for the duplicated region of the proband, we concluded that the duplicated region of chromosome 15 of the proband was inherited from her father. Some of the genes involved in the chromosome rearrangements of the proband and her mother are listed in Table 1. The proband’s mother showed severe mental retardation with speech difficulties and could not communicate. A breakpoint on chromosome 15 of the mother was found to delete part of *TCF12* (S3 Fig). During brain development, the *TCF12* gene may be responsible for expanding precursor cell populations, as demonstrated in a mouse model [28]. In addition, mutations in *TCF12* are frequent causes of coronal craniosynostosis [29], which may severely influence the development and function of the brain. It was reported by Pauline et al. that an unbalanced maternally inherited complex chromosome rearrangement in a boy with coronal craniosynostosis and intellectual disability was due to 3.64Mb heterozygous deletion of 15q21.3q22.2 region, this deletion leads to haploinsufficiency of TCF12 gene [30]. In the deletion region of chromosome 15 (chr15:57412648–59096304) of the mother, *CCNL1*, *GCOM1*, *MYZAP*, *ALDHTA2*, *AQP9*, *LIPC*, *ADAM10* and *FAM63B* were identified. *AQP9*, an aquaglyceroporin, is involved in glucose energy metabolism by facilitating glycerol diffusion in astrocytes [31]. The expression of *AQP9* in neurons also indicated that *AQP9* plays a role in energy balancing after traumatic brain injury [32]. However, as far as we know, no reports have linked *AQP9* to brain disorders in humans. The *ADAM10* gene is expressed spatially and temporally in the developing chicken spinal cord, negatively regulating neuronal differentiation during spinal cord development [33]. *ADAM10*-deficient mice die on day 9.5 of embryogenesis with defects of the developing central nervous system and cardiovascular system, among others [34]. Moreover, two rare missense mutations in *ADAM10* (Q170H and R181G) result in the increased accumulation of beta-amyloids related to Alzheimer’s disease due to disruption of the prodomain chaperone [35]. Based on these analyses, more specific studies of *AQP9* function in mental retardation should be conducted, and we plan to evaluate the amyloid level of the mother to further understand the function of *ADAM10*.

The proband demonstrated cardiac and blood vessel problems, as well as hydrocephalus and developmental retardation. Approximately one hundred genes were found in the 35.4 Mb duplicated region. It is clear that different tropomyosin isoforms (TPMs) play distinct roles in regulating cell motility, including vesicle transport, cell migration and cytokinesis [36]. TPMs move their position on the F-actin filament during contractions and are therefore considered to play an important role in the regulation of this process [37]. The proband was unable to sit alone or climb, which maybe a result of disrupted *TPM1*expression. Mutations in *TPM1* have also been linked with familial hypertrophic cardiomyopathy [38,39]. We noticed that the proband showed pulmonary hypertension, which has the potential to lead to hypertrophic cardiomyopathy; thus, we should continue to check the proband’s heart condition during the following observation period. *SMAD6* is highly expressed in the cardiac valves and outflow tract of the embryonic heart and is upregulated by shear stress [40], and two nonsynonymous variants in *SMAD6* predispose individuals to congenital cardiovascular malformation [41]. Analysis of *SMAD3* in 99 patients with thoracic aortic aneurysms and dissections and Marfan-like features revealed a heterozygous *SMAD3* mutation, as well as another 5 novel *SMAD3* mutations in 5 additional families with aneurysms-osteoarthritis syndrome [42,43]. It is known that *HCN4* is essential for proper functioning and development of the cardiac conduction system [44], and previous studies have demonstrated that *HCN4* mutations are associated with bradycardia [45,46]. However, a recent study also reported a novel *HCN4* mutation, G1097W, which was associated with atrioventricular block [47]. Considering the complex functions of *HCN4*, more experiments should be conducted to determine if there is a link between *HCN4* and the cardiac phenotype of the proband. Because we detected hydrocephalus in the proband, we considered whether several genes may have contributed to the impaired intellectual development. Cardio-facio-cutaneous (CFC) syndrome is a sporadic developmental disorder involving characteristic craniofacial features, cardiac defects (most commonly atrial septal defects and pulmonic stenosis), postnatal growth deficiency, hypotonia, and developmental delays [48]. In particular, an A-to-G transition at nucleotide 389 of the *MEK1* gene was reported in a CFC patient [49]. Thus, several features of the proband, such as atrial septal defect and growth and mental retardation, maybe associated with *MEK1*. Several genes associated with mental retardation were also identified in the duplicated region. Vertebrates contain two Cos2-like kinesin family proteins (Kif7 and Kif27), and recent studies indicated that Kif7 is the functional homolog of Cos2 in vertebrates [50]. In addition, two foetuses with a homozygous deletion in the *KIF7* gene showed a phenotype of hydrocephaly, cleft palate and micrognathia, which was similar to the proband’s phenotype [51].

In conclusion, NGS is a useful tool for detecting precise translocation breakpoints in unbalanced rearrangements. However, specific inheritance analysis of the duplications in the proband could not be solved by NGS. In fact, two breakpoints identified by FISH were not revealed by NGS due to the low resolution rate. However, the precise structure of the derivative chromosomes could be revealed by NGS in combination with conventional techniques, although additional experiments should be performed to analyse the detailed molecular mechanisms leading to the phenotype of the proband and her mother.

## Supporting Information

S1 Fig Clinical photographs of the two cases with CCRs. (A-B) Proband at 6 months. (C) Mother of the proband.
(TIF)

S2 Fig The SNP array of chromosomes 3, 5, 8, and 11 of the mother. (A) Chromosome 5 was broken at 5q21.1, with a 0.5 Mb microdeletion on the 5q21.1 region from chr5: 101,662,158–102,172,751. (B) The break regions of chromosome 11 were at 11p11 and 11q11. (C) and (D) No significant copy number variations were observed on chromosomes 3 and 8. Arrowheads show the specific microdeletion on 5q21.1 and break regions at 11p11 and 11q11.
(TIF)

S3 Fig CCRs of the mother. In total, 10 breakpoints were identified on chromosomes 3, 5, 8, 11, and 15. Deletion1 and Deletion2 were located on chromosomes 15 and 5, respectively. Black arrowheads explicitly delineate the arrangements between different chromosomes. Green arrowheads demonstrate the arrangements not identified by PCR.
(TIF)

S1 File Supplementary materials. Supplementary methods.
(DOCX)

S1 Table Primer sequences for junction fragments. (DOC)
