# Supplementary figures and images for: Mapping Breakpoints of Complex Chromosome Rearrangements Involving a Partial Trisomy 15q23.1-q26.2 Revealed by Next Generation Sequencing and Conventional Techniques
Source: PLoS One. 2016 May 24;11(5):e0154574. doi: 10.1371/journal.pone.0154574 (PMC4878739; doi:10.1371/journal.pone.0154574)

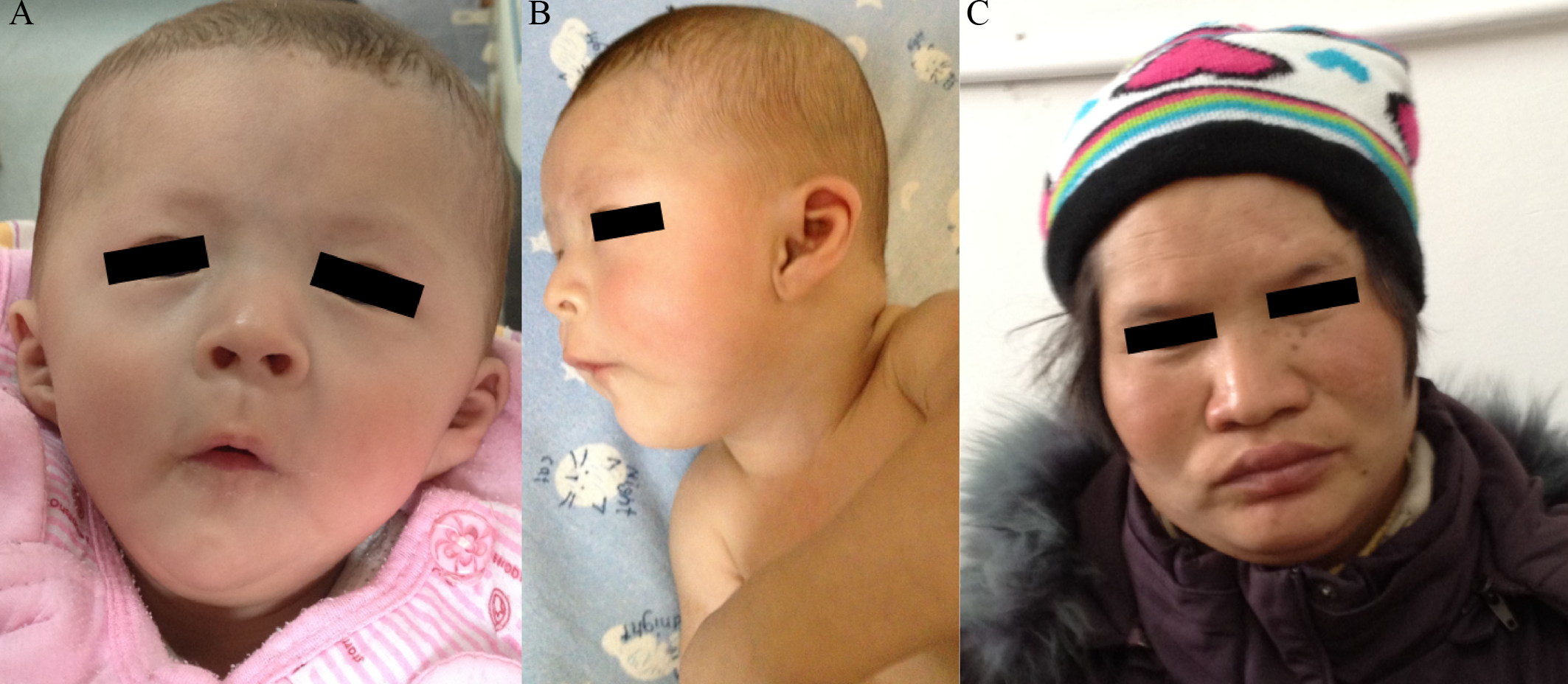

Supplement: S1 Fig — (A-B) Proband at 6 months. (C) Mother of the proband. (TIF) [file pone.0154574.s001.tif]

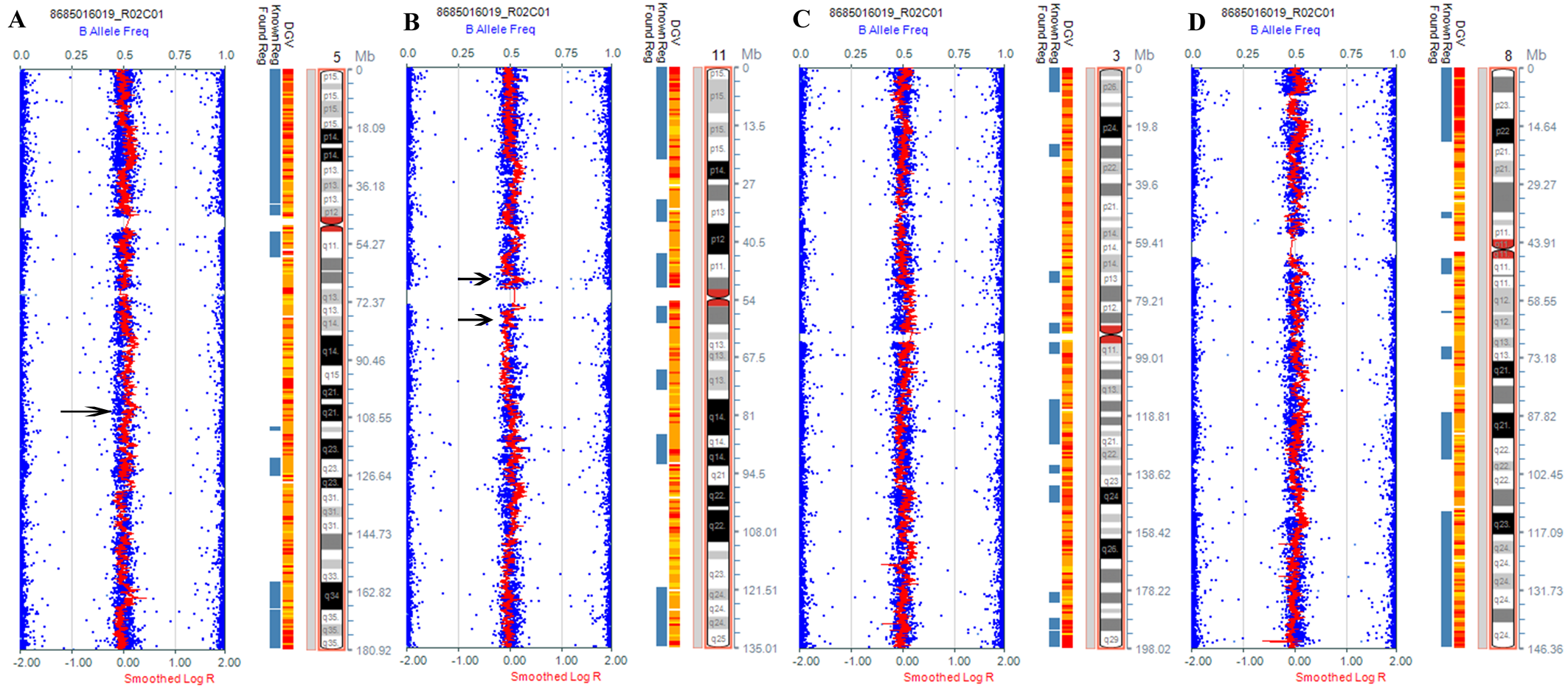

Supplement: S2 Fig — (A) Chromosome 5 was broken at 5q21.1, with a 0.5 Mb microdeletion on the 5q21.1 region from chr5: 101,662,158–102,172,751. (B) The break regions of chromosome 11 were at 11p11 and 11q11. (C) and (D) No significant copy number variations were observed on chromosomes 3 and 8. Arrowheads show the specific microdeletion on 5q21.1 and break regions at 11p11 and 11q11. (TIF) [file pone.0154574.s002.tif]

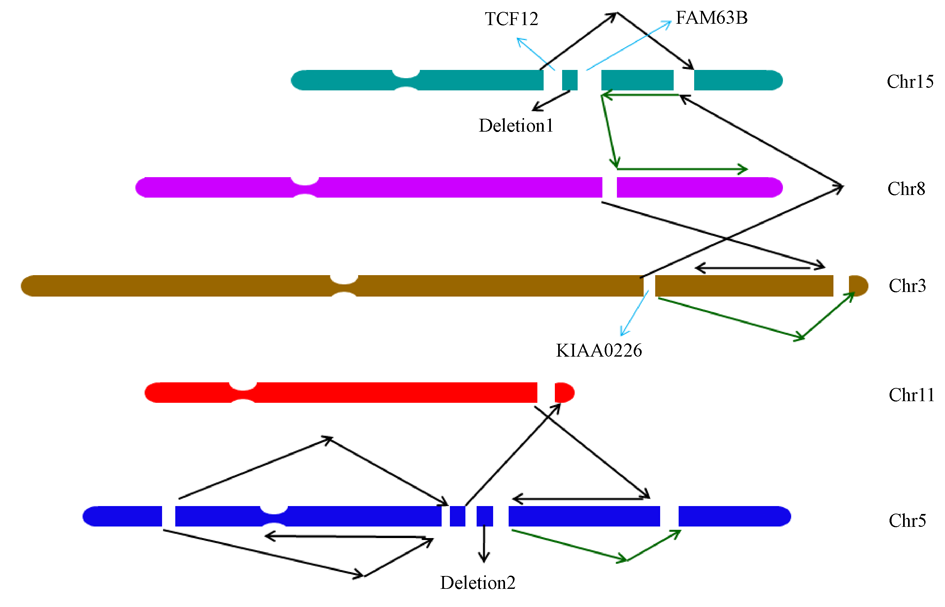

Supplement: S3 Fig — In total, 10 breakpoints were identified on chromosomes 3, 5, 8, 11, and 15. Deletion1 and Deletion2 were located on chromosomes 15 and 5, respectively. Black arrowheads explicitly delineate the arrangements between different chromosomes. Green arrowheads demonstrate the arrangements not identified by PCR. (TIF) [file pone.0154574.s003.tif]
