# Supplementary material for: Mapping Breakpoints of Complex Chromosome Rearrangements Involving a Partial Trisomy 15q23.1-q26.2 Revealed by Next Generation Sequencing and Conventional Techniques
Source: PLoS One. 2016 May 24;11(5):e0154574. doi: 10.1371/journal.pone.0154574 (PMC4878739; doi:10.1371/journal.pone.0154574)
Supplement: S1 File — Supplementary methods. (DOCX) [file pone.0154574.s004.docx]

**S1 File. Supplementary materials**

**Supplementary methods**

**Construction and Sequencing**

Genomic DNA 3 μg was fragmented by sonication to a mean length of 500 bp. Fragments within a 100 bp range (~400 to ~600 bp) were isolated from polyacrylamide gel and recovered by QiaQuick column purification (Qiagen). Fragmented DNA was performed end-repair to create non-phosphorylated blunt termini. The end-repaired gDNA fragments were then ligated to adaptor1 (Ad1). AMpure-purified Ad1-ligated DNA was subjected to PCR. This process resulted in selective amplification of the template containing both left and right Ad1 arms. The fragments were then incubated to methylate the left Ad1 arm Acul sites as well as genomics Acul sites. Ampure-purified, methylated DNA was to form monomeric dsDNA circles, containing double-stranded, unmethylated right Ad1 Acul sites. Ad1 circles were digested to form linear dsDNA structures containing Ad1 flanked by two segments of insert DNA. In a similar way as above mentioned, adaptor2 was ligated and form Ad1+2-containing dsDNA circles. The high-quality library was completed, which was loaded onto flow slides which will be read by Complete Genomics Analysis Platform of BGI. Each subject was sequenced 1 lane about 5X.

**Alignment and Data Filtering**

After removing reads containing sequencing adaptors and low quality reads with more than 2 constant unknown bases (N), the high quality pair-end reads were aligned to the NCBI human reference genome (NCBI, [GRCh37.1](https://lists.soe.ucsc.edu/pipermail/genome/2012-March/028590.html), here after called hg19) using TeraMap (in-house software) with parameters: --max-cost="5,5", --min-ls-excess-bases="7", --local-search-cost="5,5", --min-ls-call-count="12", --score-threshold="0", --no-call-limit="3", --match-buffer-size="80", --max-output="27", --mate-gap-range="0,700", --enable-local-search-if-consistent="1", --enable-multi-read-local-search="0", --max-index-matches="40", --neglected-small-gap-fraction="0.001". Only unique reads were remained in following analysis.

Detection of translocation

Our assumption is that either balanced or unbalanced translocations should be demonstrated by chimeric read pairs (paired-end reads that mapped to two different chromosomes with the expected strand orientations, unique mapping, less mismatches and higher mapping quality). These chimeric read pairs suggested possible candidate translocation “clusters” throughout the genome. The chimeric read pairs were stored in the Single file. Only this Single file of each sample was used as input for our in-house software (23) for independent translocation discovery, respectively. In general, 4 main steps were needed in the pipeline:

1) Clusters generation: Firstly, the mapped pair-ended reads that span 2 different chromosomes would be extracted and all of them were divided into groups in term of their mapped chromosomes (hg19). Secondly, the mapped reads in one chromosome (named Read1) of each group were sorted by their mapped location (hg19) and each 2 clusters were distinguished with numbered flags by a chimeric distance (default setting: 10Kbp). Eventually, the locations of the corresponding paired mapped reads (named Read 2) in the other chromosome of each cluster would be taken into consideration and the outliers which far from the statistic mode of the certain cluster would be discarded.

2) Filtering by linear correlation: Linear correlation between the locations of Read1s and Read2s was performed for each cluster and the one of which Spearman’s rank correlation coefficient less than 0.8 would be discarded. The distributions of locations of Read1s and Read2s of each cluster were supposed to follow Possion Distribution. Since that, Read1s’ corrected standard deviation (SD) and Read2s’ were calculated for comparison by the remained paired reads after removing the outliers (P<0.05), respectively. Afterwards, the putative breakpoint regions located in the flanking region (500 Kbp) of N region (unidentified in hg19) would be marked simultaneously.

3) Filtering by a cohort of control data: whole genome low-depth data (mean depth of 2~5X) of 208 persons from Chinese population were selected as control and each of them was processed by the same pipeline described above. The set of putative breakpoint regions were discarded when more than 11 samples (5%) from the control cohort harboring the putative ones either in the upstream region or downstream (5Kbp from the mean of putative breakpoint region).

4) Filtering by the same strand orientation: The clusters with the same strand orientation (++/-- or +-/-+) reads supported will be remained if they are used for balanced translocation detection. Reads supporting the translocation events with the same-side arm of chromosomes always align to the strands (++/--) whereas the strand orientations for reads supporting the translocation events with the diff-side arm of chromosomes will be combined (+-/-+). When it is used for unbalanced translocation breakpoint region detection, copy-number variations (CNV) detection will also contribute to define the breakpoint region of the derivative chromosome.
