# Supplementary material for: Mapping Breakpoints of Complex Chromosome Rearrangements Involving a Partial Trisomy 15q23.1-q26.2 Revealed by Next Generation Sequencing and Conventional Techniques
Source: PLoS One. 2016 May 24;11(5):e0154574. doi: 10.1371/journal.pone.0154574 (PMC4878739; doi:10.1371/journal.pone.0154574)
Supplement: S1 Table — (DOC) [file pone.0154574.s005.doc]

**Table S1 Primers sequences for junction fragments.**

|  | Primers | |
| --- | --- | --- |
| Name | Forward primer | Reverse primer |
| Chr11-5r | CTGTGCTTTCCCCAATGAGT | GCACCATTATAACCATGCAGC |
| Chr15-15 | CACCTCTTTCTCTTAAGCTTTCCC | ATAGGGGCAAATCGATAAAACCAA |
| Chr5-5r | TGGTCTAAGTCCCAGCTTCG | TGACCCTGAATCCTAATATCCCA |
| Chr5r-5 | TGTAAGACCAGCCAGCCTAG | GTTTCCAAGACAGAGCGTGG |
| Chr3-15r | TGGGCTCGATTTCTTGTTCAC | AGGAGAAGAATTTGCTGCGTG |

Chr11-5r for region 5 of chromosome 11 and region 5 of chromosome 5, Chr15-15 for region 1 and region 4 of chromosome 15, Chr5r-5 for region 1 and region 2 of chromosome 5, Chr5r-5 for region 2 and region 3 of chromosome 5, Chr3-15r for region 1 of chromosome 3 and region 3 of chromosome 15. The specific region were shown in Figure 5.
